# Changes in the Molecular and Functional Phenotype of Bovine Monocytes during *Theileria parva* Infection

**DOI:** 10.1128/IAI.00703-19

**Published:** 2019-11-18

**Authors:** Reginaldo G. Bastos, Kelly Sears, Kelcey D. Dinkel, Donald P. Knowles, Lindsay M. Fry

**Affiliations:** a Department of Veterinary Microbiology & Pathology, College of Veterinary Medicine, Washington State University, Pullman, Washington, USA; b Veterinary Clinical Sciences, College of Veterinary Medicine, Washington State University, Pullman, Washington, USA; c Animal Disease Research Unit, Agricultural Research Service, U.S. Department of Agriculture, Pullman, Washington, USA; University of Pennsylvania

**Keywords:** *Theileria parva*, monocytes, monocyte phenotype, monocyte function, bovine, correlate of protection, function, innate immunity, intermediate, phenotype

## Abstract

Theileria parva is the causative agent of East Coast fever (ECF), a tick-borne disease that kills over a million cattle each year in sub-Saharan Africa. Immune protection against T. parva involves a CD8^+^ cytotoxic T cell response to parasite-infected cells. However, there is currently a paucity of knowledge regarding the role played by innate immune cells in ECF pathogenesis and *T. parva* control. Here, we demonstrate an increase in intermediate monocytes (CD14^++^ CD16^+^) with a concomitant decrease in the classical (CD14^++^ CD16^−^) and nonclassical (CD14^+^ CD16^+^) subsets at 12 days postinfection (dpi) during lethal infection but not during nonlethal *T. parva* infection. *Ex vivo* analyses of monocytes demonstrated upregulation of interleukin-1 beta (IL-1β) and tumor necrosis factor alpha (TNF-α) mRNA and increased nitric oxide production during *T. parva* lethal infection compared to nonlethal infection at 10 dpi. Interestingly, no significant differences in peripheral blood parasite loads were observed between lethally and nonlethally infected animals at 12 dpi. *In vitro* stimulation with *T. parva* schizont-infected cells or Escherichia coli lipopolysaccharide (LPS) resulted in significant upregulation of IL-1β production by monocytes from lethally infected cattle compared to those from nonlethally infected animals. Strikingly, monocytes from lethally infected animals produced significant amounts of IL-10 mRNA after stimulation with *T. parva* schizont-infected cells. In conclusion, we demonstrate that *T. parva* infection leads to alterations in the molecular and functional phenotypes of bovine monocytes. Importantly, since these changes primarily occur in lethal infection, they can serve as biomarkers for ECF progression and severity, thereby aiding in the standardization of protection assessment for *T. parva* candidate vaccines.

## Abstract (precis)

Theileria parva is the causative agent of East Coast fever (ECF), a tick-borne disease that kills over a million cattle each year in sub-Saharan Africa. Immune protection against T. parva involves a CD8^+^ cytotoxic T cell response to parasite-infected cells. However, there is currently a paucity of knowledge regarding the role played by innate immune cells in ECF pathogenesis and *T. parva* control.

## INTRODUCTION

Theileria parva is a tick-borne, apicomplexan hemoparasite that causes East Coast fever (ECF), an economically important disease that kills over a million cattle each year in eastern, central, and southern Africa (1). Control of ECF is currently based on the use of acaricides to limit tick infestation and on the infection and treatment method (ITM) of immunization, in which cattle are injected with ground T. parva-infected ticks and cotreated with long-acting oxytetracycline. The use of acaricides is restricted by expense, equipment availability, environmental regulations, and the development of resistance by tick populations (2). Although ITM consistently elicits immune protection from ECF, its labor- and animal-intensive production cycle, coupled with the requirement for antibiotic cotreatment and liquid nitrogen cold storage of vaccine doses, creates significant cost and logistical barriers to widespread use. Furthermore, ITM-immunized cattle become lifelong carriers of some strains of *T. parva*, which poses a risk of disease spread (3–5). Thus, the development of novel approaches to control *T. parva* is urgently needed. To achieve this goal, it is critical to obtain a better understanding of ECF pathogenesis and the immune mechanisms involved in *T. parva* protection and susceptibility.

Clinically, ECF is characterized by marked peripheral lymphadenopathy, fever, thrombocytopenia, and leukopenia (6, 7). As the disease progresses, acutely infected animals develop pulmonary edema and pleural effusion, which results in death. We recently demonstrated that respiratory failure and death in ECF are the result of systemic activation of alveolar macrophages and consequent histiocytic vasculitis (7). Although it is well established that immune protection against *T. parva* requires the development of cytotoxic CD8^+^ T cells (8, 9), the innate immune response to the infection and its potential role in the development of either protective adaptive immunity or severe pathology remain largely uncharacterized.

Myeloid lineage cells, including monocytes, macrophages, and dendritic cells (DC), play a crucial role in the innate immune response and in skewing adaptive immune mechanisms to control diseases (10, 11). Since monocytes are progenitors of DC and macrophages, they are essential effectors of the innate immune system (12). Initially considered a homogeneous population, monocytes have recently been classified into subsets based on the expression of the surface markers CD14 and CD16 (13). Bovine monocytes have been classified into classical (CD14^++^ CD16^−^), intermediate (CD14^++^ CD16^+^), and nonclassical (CD14^+^ CD16^+^) subsets (14–16). Although monocyte phenotypes are somewhat conserved among mammalian species, the role played by each subset in infection and inflammation is controversial and often varies between different pathogen systems (15–18). Therefore, a more comprehensive picture is needed to clarify the functional aspects of different monocyte subsets in the immune response to relevant pathogens and the implications of these findings in health and disease. As an intracellular hemoprotozoan parasite, *T. parva* provides an interesting model to investigate innate immune mechanisms, including monocyte function, for related hemoparasitic infections, including Babesia spp. and Plasmodium spp.

In this study, we hypothesized that *T. parva* infection induces changes in the molecular and functional phenotypes of bovine monocytes, resulting in an alteration of cell activation. To test this hypothesis, we evaluated phenotypic and functional changes in monocytes from cattle (Bos taurus) during *T. parva* lethal infection (inoculation with parasite stabilate) and nonlethal infection (inoculation with parasite stabilate plus long-acting oxytetracycline). Taken together, the results presented in this study demonstrate alterations of monocytes during *T. parva* infection with implications for ECF pathogenesis and disease progression.

## RESULTS

### *T. parva* infection alters monocyte subset proportions in cattle.

**FIG 1:**
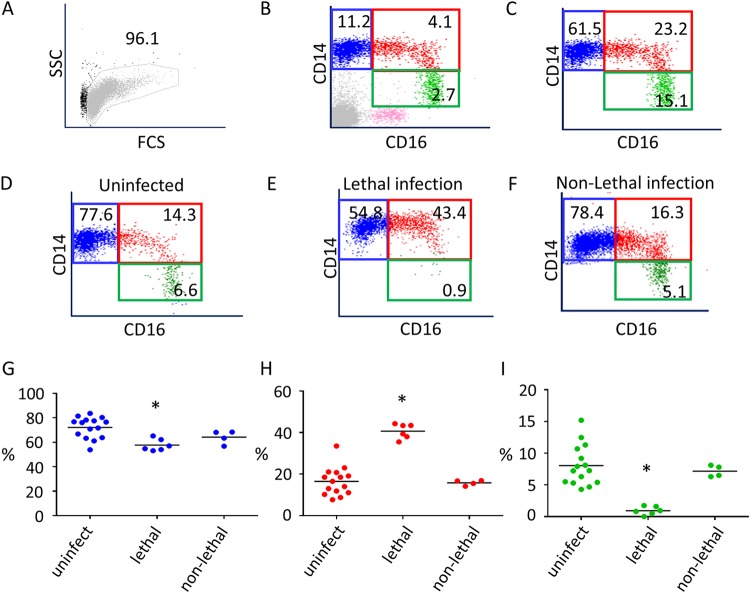
Gating strategy for the identification of monocyte subsets in cattle and proportions of monocyte populations in *T. parva*-infected animals. (A) PBMC were gated according to their complexity (side scatter [SSC]) and size (forward scatter [FSC]). Log_10_ was used on the *y* axis (SSC) and a linear scale used on the *x* axis (FCS). (B) Dot plot depicting the three monocyte subsets in total PBMC, as follows: classical CD14^++^ CD16^−^ (blue), intermediate CD14^++^ CD16^+^ (red), and nonclassical CD14^+^ CD16^+^ (green). In addition, NK cells are shown in pink, and the identity of this population was verified by the expression of CD16 and CD335 (data not shown). (C) Dot plot showing classical (blue), intermediate (red), and nonclassical (green) monocytes. (D to F) Dot plots showing the relative proportions of monocyte subsets in representative uninfected, *T. parva* lethally infected, and *T. parva* nonlethally infected cattle. For dot plots shown in panels B to F, log_10_ was used on both the *x* and *y* axes, and the numbers indicate the percentage of each monocyte subset. (G to I) Frequency of monocyte subsets in uninfected (uninfect, *n* = 15), *T. parva* lethally infected (lethal, *n* = 6), and *T. parva* nonlethally infected (non-lethal, *n* = 4) cattle. Bars indicate the mean percentage of each monocyte population. Monocyte subset proportions were compared using analysis of variance (ANOVA) and Tukey’s *post hoc* test. *, *P* < 0.05.

Due to the importance of classical, intermediate, and nonclassical monocytes in the early events of the innate immune response, it was of interest to investigate the relative subset proportions during lethal and nonlethal *T. parva* infection in cattle. In order to investigate monocyte subsets, peripheral blood mononuclear cells (PBMC) were isolated from blood using Histopaque (Sigma) and utilized for flow cytometric analyses. The cells were gated according to their complexity (side scatter [SSC]) and size (forward scatter [FSC]) to exclude cell debris and granulocytes (Fig. 1A). FSC-height versus FSC-area analysis was performed to identify and eliminate doublets from further cell analysis (data not shown). Gates to define the monocyte subsets were set using one-, two-, and three-color panels and fluorescence-minus-one controls (Fig. 1B and C). Monocyte subsets were defined and color-coded as follows: classical monocytes (CD14^++^ CD16^−^), blue; intermediate monocytes (CD14^++^ CD16^+^), red; and nonclassical monocytes (CD14^+^ CD16^+^), green (Fig. 1B and C). In addition, CD335^+^ NK cells (data not shown), also CD16^+^, are shown in pink (Fig. 1B) and were excluded from subsequent analysis of monocyte subsets.

Next, we investigated the effect of *T. parva* infection on the relative proportion of each monocyte subset. The percentage of each monocyte subset in representative uninfected, lethally infected (12 days postinfection [dpi]), and nonlethally infected (12 dpi) cattle is presented in Fig. 1D to F, respectively. As an established condition for this study, inoculation with the *T. parva* Muguga strain sporozoite stabilate Pullman 2015/4 was considered a lethal infection, whereas simultaneous inoculation of the parasite stabilate and treatment with long-acting oxytetracycline was defined as nonlethal infection. The proportion of each monocyte subset in uninfected (*n* = 15), lethally infected (*n* = 6), and nonlethally infected (*n* = 4) cattle is shown Fig. 1G to I. Monocyte phenotype was evaluated at 12 dpi in all infected cattle. At this time point, lethally infected cattle showed a significant decrease (*P* < 0.05) in classical and nonclassical monocyte subsets compared to those in nonlethally infected and uninfected animals (Fig. 1G and I). In contrast, the percentage of intermediate monocytes increased significantly (*P* < 0.05) during lethal infection compared to that observed in monocytes from uninfected cattle and those undergoing nonlethal infection (Fig. 1H). To assess the potential role of oxytetracycline treatment in the observed monocyte subset changes, two age-matched Holstein-Friesian steer calves were treated with long-acting oxytetracycline without *T. parva* infection, and monocyte subset proportions were assessed at 3, 6, 9, and 12 days later. No significant changes in the monocyte subset proportion were observed (see Fig. S1 in the supplemental material). Taken together, these results demonstrate that lethal *T. parva* infection alters monocyte subset proportions in cattle, characterized by a decrease in classical and nonclassical populations and concomitant increase in the intermediate subset.

### Expansion of intermediate monocytes is associated with progression of lethal *T. parva* infection.

**FIG 2:**
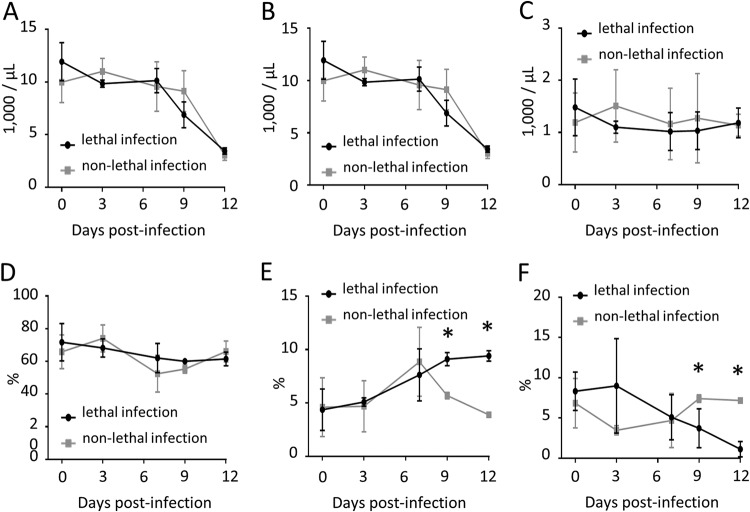
Total white blood cell counts and kinetics of changes in the proportion of classical, intermediate, and nonclassical monocytes during lethal and nonlethal *T. parva* infection of cattle. (A to C) Absolute numbers of total white blood cells (A), lymphocytes (B), and monocytes (C) in peripheral blood of lethally and nonlethally infected animals. Leukocyte counts were performed using a ProCyte Dx hematology analyzer (Idexx). (D to F) Percentages of classical (D), intermediate (E), and nonclassical (F) monocytes in PBMC from lethally and nonlethally infected cattle throughout acute infection. Averages of subset percentages were compared using Student's *t* test. *, *P* < 0.05.

After characterizing the three subsets of bovine monocytes and demonstrating that *T. parva* infection induces alterations in their relative proportions at 12 dpi, we next compared lethally and nonlethally infected cattle over time to investigate changes in the kinetics of surface expression of CD14 and CD16 on the monocyte subsets. Infected cattle were examined at 3, 7, 9, and 12 dpi. Complete blood cell counts showed a dramatic decrease in the absolute numbers of leukocytes (Fig. 2A), including lymphocytes (Fig. 2B), in whole blood during both lethal and nonlethal infection. However, no significant changes in the absolute number of total monocytes were observed (Fig. 2C). Changes in the kinetics of monocyte subset proportions were evaluated by flow cytometry, as described above (Fig. 2D to F). Analysis revealed a marked decrease in nonclassical monocytes and simultaneous increase in the intermediate subset at 9 and 12 dpi in lethally infected cattle compared to those in nonlethally infected animals (Fig. 2E and F). Interestingly, after day 7 postinfection, the percentage of nonclassical monocytes continued to decrease in lethally infected, but not nonlethally infected, animals (Fig. 2F). Also, after 7 dpi, the percentage of intermediate monocytes continued to increase in lethal, but not nonlethal, *T. parva* infection (Fig. 2E). These changes corresponded to a rapid clinical decline of lethally infected animals from 7 dpi onward, with all lethally infected cattle requiring euthanasia due to respiratory distress by day 14 postinfection.

In nonlethally infected animals, the proportion of both intermediate and nonclassical monocyte subsets at 12 dpi was similar to preinfection levels. In addition, from days 3 to 12 postinfection, the frequency of classical monocytes dropped from ∼70% to ∼60% in both lethally and nonlethally infected animals, and no significant differences were observed (Fig. 2D). Collectively, these data demonstrate that an increase in intermediate monocytes and concomitant decrease in nonclassical monocytes correlate with terminal progression of lethal *T. parva* infection.

### *T. parva* infection induces differential expression of MHC-II and CD163 in monocyte subsets.

**FIG 3:**
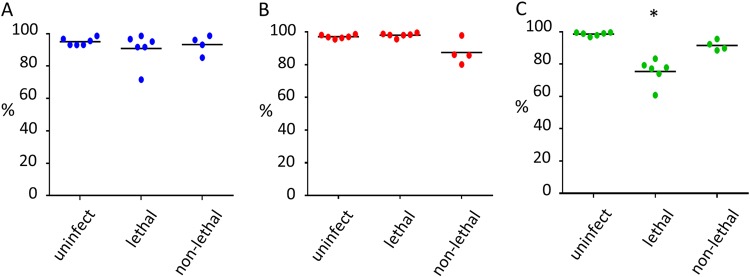
Differential expression of MHC-II by bovine monocyte subsets during *T. parva* infection. (A to C) Percentages of MHC-II expression on classical (CD14^++^ CD16^−^) (A), intermediate (CD14^++^ CD16^+^) (B), and nonclassical (CD14^+^ CD16^+^) (C) monocyte subsets in uninfected (uninfect, *n* = 6), *T. parva* lethally infected (lethal, *n* = 6), and *T. parva* nonlethally infected (non-lethal, *n* = 4) cattle. Percentages of MHC-II-positive monocytes were compared using ANOVA and Tukey’s *post hoc* test. *, *P* < 0.05. Bars indicate the mean percentage of each monocyte population.

**FIG 4:**
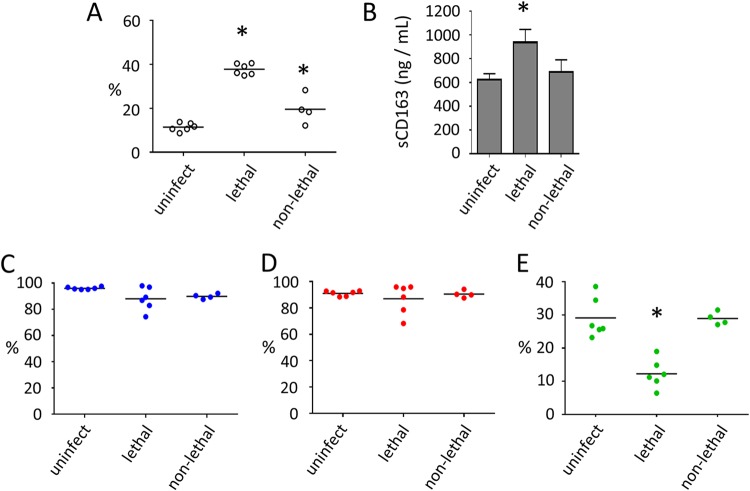
*T. parva* infection alters the expression of CD163 on the surface of bovine PBMC and monocyte subsets and the levels of soluble CD163 (sCD163) in sera from infected cattle. (A) Percentages of CD163^+^ cells in PBMC of uninfected (uninfect, *n* = 6), *T. parva* lethally infected (lethal, *n* = 6), and *T. parva* nonlethally infected (non-lethal, *n* = 4) cattle. (B) Amount (in nanograms per milliliter) of soluble CD163 (sCD163) in peripheral blood of uninfected (uninfect, *n* = 6), *T. parva* lethally infected (lethal, *n* = 6), and *T. parva* nonlethally infected (non-lethal, *n* = 4) cattle. (C to E) Percentages of CD163^+^ cells within classical (C), intermediate (D), and nonclassical (E) subsets in PBMC of uninfected (uninfect, *n* = 6), *T. parva* lethally infected (lethal, *n* = 6), and *T. parva* nonlethally infected (non-lethal, *n* = 4) cattle. CD163 surface expression and serum sCD163 levels in uninfected and infected animals were compared using ANOVA and Tukey’s *post hoc* test. *, *P* < 0.05. Bars in panels A and C to E indicate the mean percentage of each monocyte population.

To address the question of whether *T. parva* modulates the expression of effector molecules in monocytes, we investigated monocyte surface expression of major histocompatibility complex class II (MHC-II) and CD163 and levels of soluble CD163 in sera from cattle undergoing lethal and nonlethal *T. parva* infection. PBMC were isolated as described above, and surface expression of MHC-II and CD163 of each monocyte subset was assessed by flow cytometry at 12 dpi (Fig. 3 and 4). The results revealed significant (*P* < 0.05) downregulation of MHC-II on nonclassical monocytes during lethal *T. parva* infection compared to the level observed in monocytes from uninfected and nonlethally infected animals (Fig. 3C). No significant difference in MHC-II expression was detected between the classical and intermediate subsets of lethally and nonlethally infected and uninfected animals (Fig. 3A and B).

Next, we evaluated the expression of CD163 on the surface of total PBMC and levels of soluble CD163 in sera from lethally and nonlethally *T. parva* infected animals at 12 dpi (Fig. 4A and B). The results demonstrated that both lethally and nonlethally *T. parva* infected animals exhibited significantly increased (*P* < 0.05) surface CD163 expression in PBMC compared to uninfected animals (Fig. 4A). In addition, we showed that PBMC from lethally infected cattle expressed significantly higher (*P* < 0.05) levels of CD163 than PBMC from nonlethally infected animals (Fig. 4A). Furthermore, serum from lethally infected animals contained significantly higher (*P* < 0.05) levels of soluble CD163 than did serum from nonlethally infected and uninfected animals (Fig. 4B). Next, we evaluated the expression of CD163 on the surface of each monocyte subset during lethal and nonlethal *T. parva* infection. The data showed no significant differences in the surface expression of CD163 in classical and intermediate monocytes during lethal and nonlethal infection compared to that in uninfected control animals (Fig. 4C and D). In contrast, nonclassical monocytes from lethally infected cattle exhibited significantly lower (*P* < 0.05) surface CD163 expression than did those from nonlethally infected and uninfected individuals (Fig. 4E). Together, these data demonstrate that *T. parva* infection alters the expression of MHC-II and CD163 on monocytes and the levels of soluble CD163 in sera. The observed changes in MHC-II and CD163 expression were substantially more pronounced during lethal infection and primarily affected the nonclassical monocyte subset.

### Lethal *T. parva* infection leads to upregulation of monocyte proinflammatory mediators.

**FIG 5:**
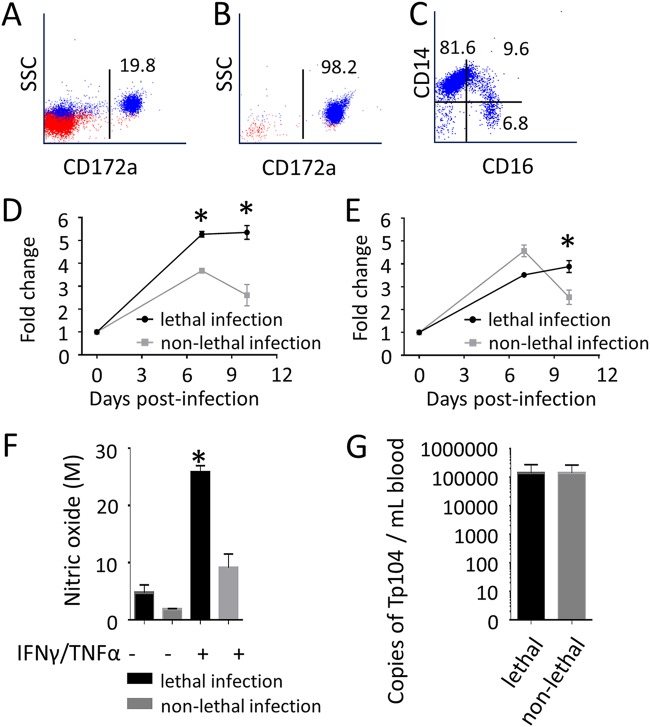
*T. parva* upregulates the production of inflammatory mediators by monocytes during lethal infection. (A) Percentage of CD172a^+^ cells in PBMC of an uninfected, representative steer. (B) Positive selection of CD172a^+^ cells from PBMC of an uninfected, representative steer. (C) CD172a^+^ cells include the classical (CD14^++^ CD16^−^), intermediate (CD14^++^ CD16^+^), and nonclassical (CD14^+^ CD16^+^) monocyte subsets. (D and E) Expression of mRNA for IL-1β (D) and TNF-α (E) in CD172a^+^ cells from *T. parva* lethally infected (*n* = 6) and nonlethally infected (*n* = 4) cattle. Gene expression levels were compared using Student's *t* test. (F) Production of nitric oxide (NO) in CD172a^+^ cells from *T. parva* lethally infected (*n* = 6) and nonlethally infected (*n* = 4) cattle following 96 h of exposure to exogenous recombinant bovine IFN-γ (50 U/ml; Ciba-Geigy) plus recombinant human TNF-α (2,500 U/ml; R&D Systems). NO production was compared using ANOVA and Tukey’s *post hoc* test. (G) *T. parva* peripheral blood parasite load, determined by quantitative PCR, in *T. parva* lethally (*n* = 6) and nonlethally (*n* = 4) infected cattle. Parasite loads were compared using Student's *t* test. *, *P* < 0.05.

We next investigated interleukin-1 beta (IL-1β) and tumor necrosis factor alpha (TNF-α) expression and nitric oxide (NO) production by monocytes during lethal and nonlethal *T. parva* infection. In order to evaluate the entire monocyte population and assess all three subsets simultaneously, we first performed a positive selection of CD172a^+^ cells. By colabeling CD172a^+^ selected cells with anti-CD14 and anti-CD16 antibodies, we demonstrated that the CD172a^+^ population contains the classical, intermediate, and nonclassical monocyte subsets (Fig. 5A to C). Subsequently, we evaluated the kinetics of IL-1β and TNF-α expression in CD172a^+^ monocytes during lethal and nonlethal *T. parva* infection using reverse transcriptase quantitative PCR (RT-qPCR). The results demonstrated significant upregulation (*P* < 0.05) of IL-1β mRNA at 7 and 10 days after lethal infection compared to the level after nonlethal infection (Fig. 5D). Also, TNF-α mRNA was significantly upregulated (*P* < 0.05) in lethally infected cattle at 10 dpi compared to the level in nonlethally infected animals (Fig. 5E). To evaluate the ability of monocytes to produce NO, CD172a^+^ cells from lethally and nonlethally infected animals were exposed to exogenous recombinant bovine gamma interferon (IFN-γ) plus recombinant bovine TNF-α. The results demonstrated higher (*P* < 0.05) NO production by monocytes from lethally infected cattle than by those from nonlethally infected animals (Fig. 5F). Strikingly, despite alterations in cytokine mRNA levels and NO production, no significant difference in peripheral blood parasite loads was detected at 12 dpi between lethal and nonlethal infections (Fig. 5G). Collectively, these data show that lethal *T. parva* infection induces significant upregulation of IL-1β and TNF-α mRNA in bovine monocytes, and that monocytes from lethally infected animals produced significantly more NO than did those from nonlethally infected cattle. Additionally, these alterations in monocyte function are independent of the parasite load in peripheral blood.

### Monocytes from *T. parva* lethally and nonlethally infected cattle respond differently to *T. parva* schizont-infected cells and Escherichia coli LPS.

**FIG 6:**
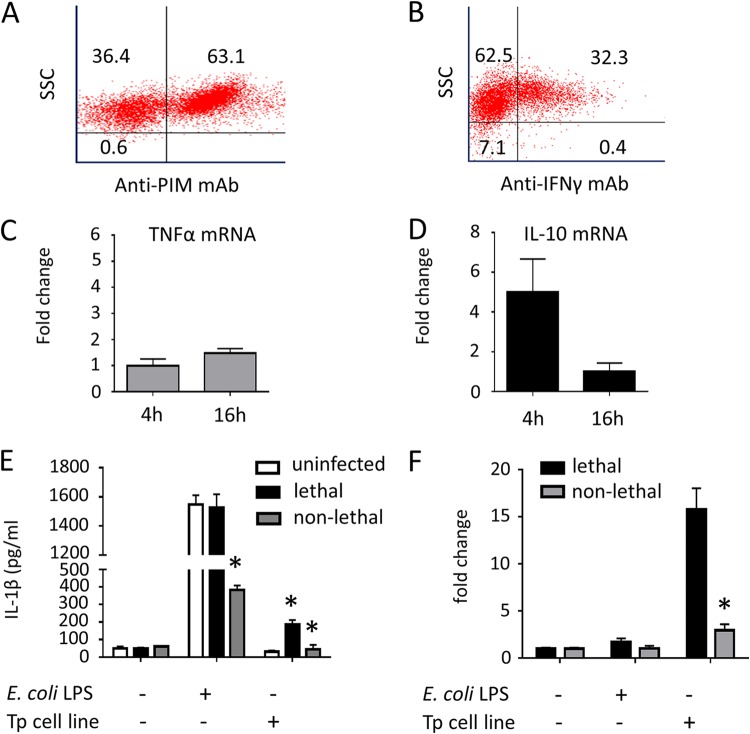
Differential response of monocytes from *T. parva* lethally and nonlethally infected cattle to *T. parva* infected cells and E. coli LPS. (A) Intracellular staining for the *T. parva* polymorphic immunodominant molecule in a representative schizont-infected cell line. (B) Intracellular staining for IFN-γ in a representative schizont-infected cell line. (C and D) Expression of mRNA for TNF-α (C) and IL-10 (D) in a representative schizont-infected cell line at 4 and 16 h after Histopaque centrifugation. (E) Levels of soluble IL-1β in CD172a^+^ cell supernatant following stimulation of monocytes from uninfected (*n* = 6), *T. parva* lethally infected (*n* = 6), and *T. parva* nonlethally infected (*n* = 4) cattle with *T. parva* schizont-infected cells (using a transwell format) or E. coli LPS (1 μg/ml). Amounts of soluble IL-1β were compared using ANOVA and Tukey’s *post hoc* test. (F) Expression of IL-10 mRNA in CD172a^+^ cells from *T. parva* lethally infected (*n* = 6) and nonlethally infected (*n* = 4) cattle following stimulation with *T. parva* schizont-infected cells (using a transwell format) or E. coli LPS (1 μg/ml). IL-10 mRNA expression was compared using ANOVA and Tukey’s *post hoc* test. *, *P* < 0.05.

To investigate potential differential activation of bovine monocytes during *T. parva* infection, cells from lethally and nonlethally infected animals were exposed *in vitro* to *T. parva* schizont-infected lymphocytes or E. coli lipopolysaccharide (LPS). Subsequently, monocyte expression of soluble IL-1β was measured using an enzyme-linked immunosorbent assay (ELISA), and IL-10 expression was assessed using RT-qPCR. It has been demonstrated that *T. parva* schizonts induce transformation and proliferation of bovine T and B cells. These infected transformed lymphocytes are essentially immortalized and can be cultured *in vitro* as long as the intralymphocytic parasites remain viable (19). Here, we investigated the effect of *T. parva* schizont-infected cells on monocytes from lethally and nonlethally infected cattle. The *T. parva* schizont-infected immortalized cell lines were established and maintained as previously described (19) and were routinely checked for the presence of *T. parva* schizonts using intracellular staining for the *T. parva* polymorphic immunodominant molecule (PIM) (Fig. 6A). We next investigated whether the *T. parva*-infected lymphocyte lines produced cytokines that could potentially modulate monocyte activation. Using intracellular staining and flow cytometry, we demonstrated that schizont-infected lymphocyte lines produced considerable amounts of IFN-γ (Fig. 6B). The schizont-infected lymphocytes also expressed detectable levels of mRNA for TNF-α and IL-10 (Fig. 6C and D).

In order to investigate monocyte activation, CD172a^+^ cells from lethally and nonlethally infected animals were positively selected and exposed to *T. parva* schizont-infected cell lines or E. coli LPS. For the schizont-infected cell stimulation, transwell culture plates were used to prevent cell-to-cell interaction between monocytes and parasite-infected cells. Following stimulation with schizont-infected cells, monocytes from both lethally and nonlethally *T. parva*-infected cattle produced significantly higher (*P* < 0.05) levels of IL-1β than did those from uninfected animals. Interestingly, monocytes from lethally infected animals produced significantly more (*P* < 0.05) IL-1β than did monocytes from nonlethally infected animals when exposed to *T. parva* schizont-infected cells (Fig. 6E). Monocytes from nonlethally infected cattle also produced significantly less (*P* < 0.05) IL-1β than did monocytes from lethally infected animals following exposure to E. coli LPS (Fig. 6E). No soluble IL-1β was detected in the supernatant from *T. parva* schizont-infected cells cultured alone (data not shown). In addition, monocytes from lethally infected animals expressed approximately 10-fold more (*P* < 0.05) IL-10 mRNA than did those from nonlethally infected animals following exposure to *T. parva* schizont-infected cells (Fig. 6F). Altogether, these data demonstrate that monocytes from lethally infected cattle respond differently to *T. parva*-infected cells and E. coli LPS than do monocytes from nonlethally infected animals. Even though lethal infection induces a marked production of the proinflammatory cytokine IL-1β by monocytes, this is contrasted by the expression of considerable amounts of mRNA for the regulatory cytokine IL-10 by these cells.

## DISCUSSION

In this study, we sought to investigate changes in the molecular and functional phenotypes of bovine monocytes during *T. parva* infection. By comparing monocytes from cattle undergoing lethal *T. parva* infection (those inoculated with parasite stabilate alone) and nonlethal infection (those inoculated with parasite stabilate and cotreated with long-acting oxytetracycline), our data demonstrate significant alterations in monocyte phenotype and function during acute infection. Furthermore, the results are consistent with dysregulated monocyte activation that is significantly more pronounced in lethally infected cattle than in nonlethally infected cattle. Altogether, the present findings support our hypothesis and demonstrate that *T. parva* infection induces changes in the molecular and functional phenotypes of monocytes, leading to altered cell activation.

Monocytes are a major component of the mononuclear phagocyte system and provide early signals to initiate the innate immune response and support the development of an adaptive immune response to a given pathogen (10). As progenitor cells for DC and macrophages, monocytes also play a critical role in inflammation and tissue homeostasis (10). In several species, including humans, nonhuman primates, and cattle (18, 20), monocytes have been classified into three subsets based on the expression of the surface markers CD14 and CD16. In cattle, the monocyte subsets have been defined as classical (CD14^++^ CD16^−^), intermediate (CD14^++^ CD16^+^), and nonclassical (CD14^+^ CD16^+^) (14–16). Using PBMC that underwent minimal *in vitro* manipulation, we were able to establish the frequency of each monocyte subset in adult uninfected cattle, which is in agreement with previous studies using bovine PBMC (13, 20).

Previous studies in humans have demonstrated phenotypical and functional alterations in monocyte populations in response to different infectious diseases (21–23). Here, we examined the effect of *T. parva* infection on the frequency of monocyte subsets in cattle. Strikingly, the progression of lethal *T. parva* infection correlated with an increase in the frequency of intermediate monocytes and a decrease in the frequency of classical and nonclassical monocytes. Expansion of intermediate monocytes has been described during bacterial, viral, and parasitic infections of humans and has been proposed as a prognostic biomarker for disease progression (20, 23–27). Here, we showed not only that nonclassical monocytes decrease during lethal *T. parva* infection, but that this subset is essentially undetectable in peripheral blood at 12 dpi. This observation is in agreement with previous findings in systemic inflammatory responses to bacterial infections in humans (15, 26). Previous studies have shown that an increase in CD16^+^ cells correlates with increased disease severity (25, 28, 29). Our data support this premise, considering the marked increase in intermediate monocytes during lethal *T. parva* infection compared to nonlethal infection and uninfected animals. Based on our results, we propose to use the expansion of intermediate CD14^++^ CD16^+^ monocytes and the concomitant decrease in nonclassical CD14^+^ CD16^+^ monocytes as a biomarker for ECF progression.

A consistent sign of ECF is leukopenia characterized by severe lymphopenia and neutropenia in the peripheral blood of *T. parva*-infected cattle (7). Our results corroborate this finding, and both lethally and nonlethally infected cattle developed leukopenia between 7 and 9 dpi. Notably, the absolute monocyte count was not significantly altered during lethal or nonlethal infection, despite changes in monocyte subset proportions. By 12 dpi, monocyte subset proportions in nonlethally infected cattle returned to preinfection physiological levels. This reinforces the usefulness of alterations in monocyte subset proportions as a biomarker for lethal *T. parva* infection and ECF progression. Such objective, consistent biomarkers of disease are crucial for the assessment of protection during postvaccination challenge studies in cattle as we strive to develop improved control measures for ECF.

Downregulation of MHC molecules on the surface of monocytes has been demonstrated during systemic inflammation and is considered the gold standard for the prognosis of sepsis-induced immunosuppression in humans (30, 31). Here, we showed downregulation of MHC-II molecules on monocytes, particularly in the nonclassical subset, during lethal *T. parva* infection. Interestingly, lethal infection also induced downregulation of CD163 on the surface of nonclassical monocytes. In addition, changes in the surface expression of CD163 correlated with increased soluble CD163 in serum samples from lethally infected animals. Considering that no significant alterations were observed in the expression of either surface-expressed or soluble CD163 during nonlethal infection, the results suggest that alterations in this molecule correlate with disease severity. Since CD163 can act as either a pro- or anti-inflammatory molecule (32–35), the role of CD163^+^ monocytes in *T. parva* infection and ECF pathogenesis remains to be determined.

Cytokine expression during *T. parva* infection has been previously investigated in cattle and African buffaloes (36, 37). The published study in cattle reported increased IL-1β and IL-6 mRNA in peripheral blood leukocytes during acute infection (36). Here, we evaluated cells positive for CD172a, a member of the signal regulatory protein family, which has been shown to be expressed on the surface of bovine monocytes (38, 39). We first verified that the CD172a^+^ cell subset includes classical, intermediate, and nonclassical monocytes and then demonstrated the upregulation of IL-1β mRNA during *T. parva* infection. In addition, we expanded previously published observations by also showing upregulation of TNF-α mRNA and increased NO production in monocytes from lethally infected cattle compared to results from nonlethally infected animals. Dysregulation in the production of proinflammatory mediators has previously been implicated in the pathogenesis of ECF (7), and the present results corroborate these findings by demonstrating functional alterations in monocytes during lethal *T. parva* infection. These data also indicate that alterations in monocyte activation are independent of the peripheral blood parasite load, suggesting that dysregulation of this innate immune cell population, rather than parasite dose alone, may play a role in ECF pathogenesis.

It has been demonstrated that myeloid cells, such as monocytes, can be polarized *in vitro* into a proinflammatory or regulatory phenotype (40, 41). We explored this characteristic and evaluated the ability of CD172a^+^ monocytes from lethally and nonlethally infected cattle to produce cytokines following the exposure to *T. parva* schizont-infected cells or E. coli LPS. In this study, we demonstrated that CD172a^+^ monocytes from lethally infected animals produced soluble IL-1β and expressed significantly increased levels of IL-10 mRNA following exposure to *T. parva* schizont-infected cells, suggesting the development of a mixed pro- and anti-inflammatory phenotype during lethal *T. parva* infection and further supporting previous studies using bovine PBMC (36, 37).

Cattle that recover from natural *T. parva* infection develop lifelong immunity against homologous and/or related parasite strains. This fact is exploited by the ITM method of immunization, which is used to control ECF in some areas of endemicity (42). In this vaccination method, animals are inoculated with a lethal dose of ground tick stabilate containing live sporozoites and are simultaneously treated with long-acting oxytetracycline to prevent the development of ECF (43). Although the molecular mechanisms underlying the control of *T. parva* by oxytetracycline remain to be determined, *in vitro* studies have shown that oxytetracycline retards the development of *T. parva* sporozoites into schizonts (44). In addition, it has been demonstrated that oxytetracycline can downregulate immune mediators of inflammation and inhibit T lymphocyte proliferation (45). Our results demonstrate that monocytes from nonlethally infected cattle treated with oxytetracycline expressed significantly less IL-1β and TNF-α mRNA and produce less NO than monocytes from lethally infected animals who are not treated with oxytetracycline. Interestingly, we found that oxytetracycline alone has no effect on monocyte subset proportions in cattle. These data, combined with the absence of significant differences in peripheral blood parasite load between lethally and nonlethally infected animals, provide further support for the idea that host innate immune mediators play an important role in ECF pathogenesis. An added effect of delayed parasite maturation cannot be ruled out, and further studies on the mechanism of oxytetracycline in preventing severe ECF, especially its effects on monocyte function, are warranted.

In conclusion, the present data demonstrate that *T. parva* infection induces alterations in monocytes that may play a role in ECF pathogenesis. Based on the results, we propose a model for the immunological events that occur in peripheral blood during *T. parva* infection. Lethal infection results in changes in monocyte subset proportions and cell phenotype, leading to alterations in cell activation, systemic immune dysregulation, and pathology, as previously shown (7). In contrast, monocytes from nonlethally infected animals showed significantly less severe phenotypic and functional alterations. We propose the use of the phenotypic and functional alterations in monocytes that occur during lethal infection as biomarkers for ECF progression.

## MATERIALS AND METHODS

### Cattle and *Theileria parva* infection.

Fifteen Holstein-Friesian steer calves (Bos taurus) were used in this study. Animals were obtained at 3 to 12 months of age, vaccinated against pathogenic Clostridium species, and confirmed *T. parva* negative using the *T. parva* PIM ELISA, as previously described (46). The animals were initially used to establish the baseline results of monocyte phenotyping and functional assays in uninfected cattle. Subsequently, six animals were infected with 1 ml of *T. parva* sporozoite stabilate (Pullman 2015/4, Muguga strain), as previously described for lethal infection (47), and four cattle were infected with the same parasite stabilate and concomitantly treated intramuscularly with 20 mg/kg of body weight of long-acting oxytetracycline (LA200; Zoetis), as previously described for nonlethal infection (48). Both lethally and nonlethally infected animals were monitored daily for fever, leukopenia, and respiratory distress. Complete blood counts were performed using a ProCyte Dx hematology analyzer (Idexx), following the manufacturer’s instructions. The infected animals were tested by real-time quantitative PCR to detect the *T. parva* 104 gene in peripheral blood using the following primers at 60°C of annealing temperature: 5′-CAGATGGAAGTGAAGTGT-3′ and 5′-TAAATGAACAAGTGATGC-3′. All lethally infected animals developed signs of respiratory distress at 13 to 14 dpi and were humanely euthanized via intravenous (i.v.) injection of sodium pentobarbital (Vortech Pharmaceuticals). As a control for the potential effects of long-acting oxytetracycline treatment, two additional age-matched Holstein-Friesian steers were treated with a single intramuscular injection of long-acting oxytetracycline (LA200), and their peripheral blood monocyte subset proportions were measured at 3, 6, 9, and 12 days postinjection, as described below. All cattle were maintained throughout the study according to protocols approved by the Washington State University Institutional Animal Care and Use Committee (protocol numbers 4975 and 4980).

### Monocyte phenotyping.

Peripheral blood was collected via jugular venipuncture into Vacutainer tubes containing acid citrate dextrose (ACD; Becton, Dickinson), and PBMC were isolated using Histopaque (Sigma), per the standard protocol. Cells were labeled with monoclonal antibodies to surface markers (Table S1), followed by secondary antibodies (Table S2) using standard flow cytometry protocols, as previously described (39, 49). Flow cytometric analysis was performed using a Guava easyCyte flow cytometer (Millipore), and data were acquired using InCyte (guavaSoft 3.1.1). One-, two-, and three-color panels were used for monocyte phenotyping. For multicolor panels, fluorescence-minus-one and single-stained controls were used to calculate compensation values. A minimum of 20,000 events were collected for each cell sample. After acquisition, the results were analyzed in FCS Express version 6 (DeNovo software). The gating strategy used to define monocyte subsets is described in the Results and presented in Fig. 1A to C. The expression of monocyte subset surface markers is presented using dot plots, where a log_10_ scale was used on both the *x* and *y* axes.

### Soluble CD163 ELISA.

Levels of soluble CD163 in serum from *T. parva*-infected and uninfected cattle were evaluated using a commercial ELISA kit, per the manufacturer’s protocol (MyBioSource). ELISA plates were read at 450 nm, and the optical density results were used to calculate the concentration of CD163 using a standard curve (MyBioSource). The results are presented as nanograms per milliliter of soluble CD163 in serum.

### Intracellular IFN-γ staining.

*T. parva* schizont-infected cells were evaluated for their ability to produce IFN-γ using intracellular staining (ICS). Briefly, *T. parva*-infected cells were centrifuged in a Histopaque gradient for 30 min at 900 × *g*, suspended in complete RPMI (cRPMI) medium, and incubated at 37°C under 5% CO_2_ for 16 h. After that, the cells were treated with Golgi Plug (Becton, Dickinson) for 4 h following the manufacturer’s protocol and subsequently treated with Cytofix/Cytoperm Plus (Becton, Dickinson), following the manufacturer’s protocol. Cells were stained with the anti-bovine IFN-γ monoclonal antibody clone CC302 (Bio-Rad), following the manufacturer’s protocol, and analyzed by flow cytometry, as described above.

### CD172a-positive selection.

CD172a^+^ monocytes were positively selected from PBMC, as previously described (39), using MACS microbeads (Miltenyi Biotec), following the manufacturer’s instructions. Briefly, PBMC were counted, suspended in MACS buffer (phosphate-buffered saline [PBS] without Ca^2+^ and Mg^2+^ [pH 7.0], supplemented with 0.5% bovine serum albumin [BSA] and 2 mM EDTA), and incubated with an anti-bovine CD172a monoclonal antibody (Table S1) at a concentration of 2 μg/10^6^ cells for 15 min at 4°C. After incubation, cells were washed twice in MACS buffer and incubated with 20 μl of goat anti-mouse IgG microbeads (Miltenyi Biotec) for 15 min at 4°C. After incubation with microbeads, cells were loaded into MACS LS columns (Miltenyi Biotec) to select the CD172a^+^ cell population. After selection, CD172a^+^ cells were suspended in complete RPMI (cRPMI; 10% fetal bovine serum, 24 mM HEPES, 2 mM l-glutamine, and 10 μg/ml gentamicin) and counted, and their purity was evaluated using flow cytometry.

### NO production.

The production of NO was determined by measuring the accumulation of nitrite, the stable oxidized form of nitric oxide, in cell culture medium using the Griess reaction. Briefly, CD172a^+^ monocytes (10^6^ cells/ml) from lethally and nonlethally *T. parva*-infected cattle were stimulated for 96 h with recombinant bovine IFN-γ (50 U/ml; Ciba-Geigy) plus recombinant human TNF-α (2,500 U/ml; R&D Systems), as previously described (39). Fifty microliters of cell culture supernatant was then transferred to a 96-well plate and a Griess reaction performed, following the manufacturer’s protocol (Promega). The experiment was repeated three times, and samples were tested in duplicate each time. The results are shown as the mean micromolar concentration of nitrite in cell culture supernatant.

### *In vitro* stimulation of CD172a^+^ monocytes.

CD172a^+^ cells were selected as described above, plated in cRPMI, and stimulated with either *T. parva* schizont-infected cells or E. coli LPS (1 μg/ml). Cocultures of CD172a^+^ monocytes (10^6^ cells/ml) and *T. parva* schizont-infected cells (10^6^ cells/ml) were performed in a transwell format using two-chamber 24-well plates with 0.4-μm pores (Costar). The use of transwell culture plates prevented cell-to-cell contact between monocytes and *T. parva* schizont-infected cells, and it allowed an evaluation of the effects of potential soluble factors from the parasite-infected cells on monocytes. The proinflammatory cytokine IL-1β was measured in the supernatant of monocytes stimulated *in vitro* with either *T. parva* schizont-infected cells or E. coli LPS for 16 h using a bovine ELISA, following the manufacturer’s protocol (Thermo Scientific). Levels of soluble IL-1β are presented as picograms per milliliter. At 16 h poststimulation, reverse transcriptase quantitative PCR (RT-qPCR) was performed to measure IL-10 mRNA levels in CD172a^+^ monocytes (see below). Additionally, *T. parva* schizont-infected cells were assessed for the level of parasite infection by intracellular staining for PIM, as previously described (50). The levels of intracellular IFN-γ (49) and mRNA for TNF-α and IL-10 were also evaluated in *T. parva* schizont-infected cells.

### Cytokine expression.

Cytokine mRNA expression was evaluated by RT-qPCR using CD172a^+^ cells. A total of 10^6^ cells were collected in 200 μl of RNA*later* (Thermo Fisher Scientific) and stored at –20°C until use. Total RNA was extracted using the Aurum Total RNA minikit (Bio-Rad), according to the manufacturer’s protocol. Two hundred nanograms of total RNA was utilized for cDNA synthesis using the iScript cDNA synthesis kit (Invitrogen), following the manufacturer’s protocol. Quantitative PCR for IL-1β, IL-10, and TNF-α was performed in a CFX96 real-time PCR detection system using the SsoFast EvaGreen supermix (Bio-Rad) and the primers described in Table S3. The cycling conditions consisted of an enzyme activation step of 95°C for 30 s, followed by 40 cycles of 95°C denaturation for 5 s and annealing/extension of 60°C for 5 s. Reactions were performed in duplicate in 20 μl using 200 nM each primer and 2 μl of a 1/20 dilution of cDNA as the template. For normalization of the cytokine RT-qPCR, bovine actin, tubulin, ATPase, 18S, HPRT1, and SDHA genes were evaluated as reference gene candidates (Table S3). GeNorm analysis (51) showed that SDHA and ATPase were the most stable genes in the *ex vivo* samples from uninfected and infected animals. Therefore, these genes were used as reference genes for the normalization of RT-qPCR (Fig. S2). RT-qPCR data were analyzed using the CFX Manager software (Bio-Rad), and gene expression was calculated using the 2^−ΔΔ^*^CT^* method (52).

### Statistical analysis.

The percentages of monocytes positive for surface expression of CD14, CD16, MHC-II, and CD163 in uninfected, lethally *T. parva* infected, and nonlethally *T. parva* infected cattle were compared using ANOVA and Tukey’s *post hoc* test using Prism version 6 (GraphPad Software). Mean monocyte subset proportions, levels of cytokine mRNA, soluble IL-1β levels, NO production, and peripheral blood parasite loads were compared with a two-tailed *t* test using Prism version 6.

## Supplementary Material

Supplemental file 1
