# Supplementary material for: Changes in the Molecular and Functional Phenotype of Bovine Monocytes during Theileria parva Infection
Source: Infect Immun. 2019 Nov 18;87(12):e00703-19. doi: 10.1128/IAI.00703-19 (PMC6867863; doi:10.1128/IAI.00703-19)
Supplement: Supplemental file 1 [file IAI.00703-19-s0001.pdf]

Table S1. Primary monoclonal antibodies used in this study.

Antibody Identification	Isotype	Specificity	Source
KD1	IgG2a	CD16	Bio-Rad
CAM36A	IgG1	CD14	WSU Monoclonal Center
CAM66A	IgM	CD14	WSU Monoclonal Center
DH59B	IgG1	CD172a	WSU Monoclonal Center
AKS1	IgG1	CD335	Bio-Rad
LND68A	IgG1	CD163	WSU Monoclonal Center
TH14B	IgG2a	MHC class II	WSU Monoclonal Center
CAT82A	IgG1	MHC class II	WSU Monoclonal Center
CC302	IgG1	Bovine IFNγ	Bio-Rad

Table S2. Secondary antibodies used in this study.

Antibody Identification	Source
Alexa Flour™ 488 goat anti-mouse IgM	Thermo Fisher Scientific
Alexa Flour™ 647 goat anti-mouse IgM	Abcam
Alexa Flour™ 488 goat anti-mouse IgG1	Thermo Fisher Scientific
Alexa Flour™ 555 goat anti-mouse IgG1	Thermo Fisher Scientific
Alexa Flour™ 647 goat anti-mouse IgG1	Thermo Fisher Scientific
Alexa Flour™ 488 goat anti-mouse IgG2a	Thermo Fisher Scientific
Alexa Flour™ 555 goat anti-mouse IgG2a	Thermo Fisher Scientific
Alexa Flour™ 647 goat anti-mouse IgG2a	Thermo Fisher Scientific

Table S3. Primers for cytokine and reference genes used in reverse transcriptase real-time quantitative PCR.

Gene	Left primer (3'-5')	Right primer (3'-5')	GenBank	Ref
IL-1β	gccttcaataactgtggaaccaat	gtatatttcaggcttggtgaaagga	M37211	(1)
IL-10	gagcaaggcggtggagaagg	gatgaagatgtcaaactcactcatgg	KX013147	(2)
TNFα	tctaccagggaggagtcttcca	gtccggcaggttgatctca	NM_173966.3	(3)
HPRT1	tgctgaggatttggagaagg	caacaggtcggcaaagaact	NM_001034035.2	(4)
SDHA	gcgcgggttgacgagta	cttcccagtcttgatgt	BT030722	(2)
Actin	tactcctggttgctgat	gtgtggattggcggct	AH001130.2	this study
Tubulin	actccatctcgtccat	caacagcacagccatc	AF035420	(2)
18S	atccaatcggtagtagc	agtaagtgcgggtcata	NR_036642.1	this study
ATPase	tccaccccctccttga	cgtcttaccaccactgc	XM_005206137.3	this study

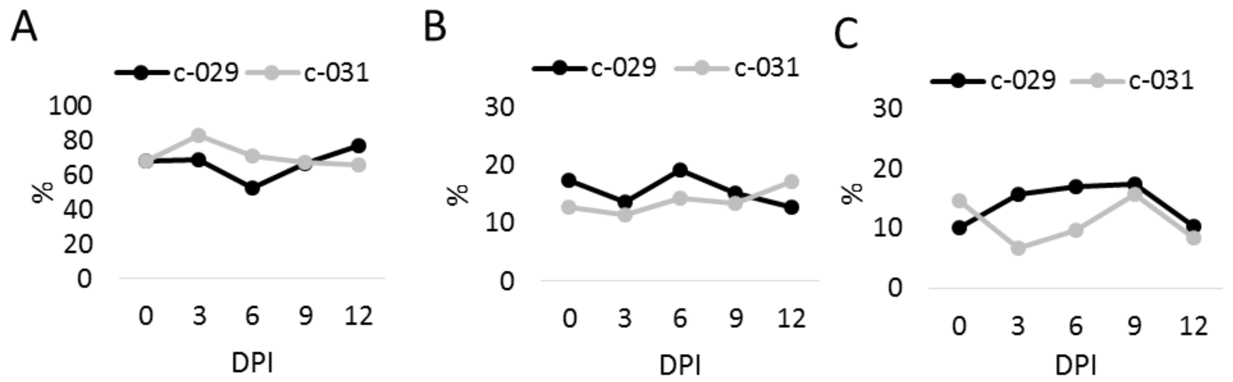

Fig S1 Proportions of classical (A), intermediate (B), and non-classical (C) monocytes in two calves (c-029 and c-031) injected intramuscularly with long-acting oxytetracycline (LA200, Zoetis) (20 mg/kg), without *T. parva* infection. The "x" axis indicates days-post injection (DPI) of oxytetracicline and the "y" axis shows the percentage of monocytes.

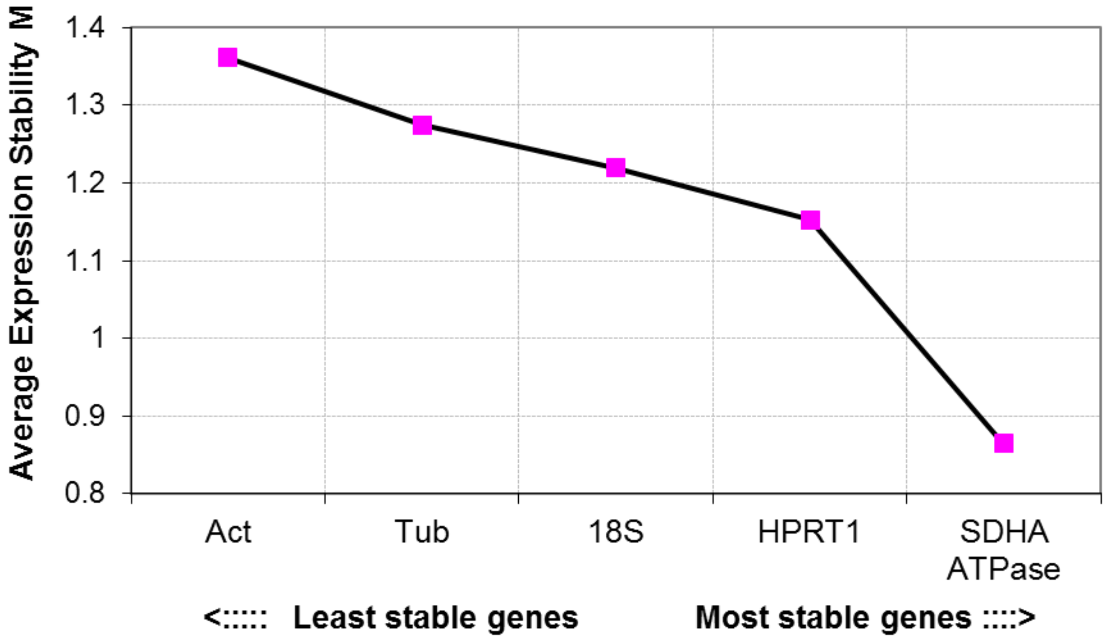

Fig S2 GeNorm analysis of bovine actin (Act), tubulin (Tub), 18S ribosomal RNA (18S), hypoxanthine phosphoribosyltransferase 1 (HPRT1), succinate dehydrogenase (SDHA)

and ATPase as potential reference genes for quantitative PCR normalization. Graph shows values of average expression stability of the reference gene candidates. cDNA from *ex vivo* monocytes from uninfected and *T. parva* infected cattle were analyzed. The most stable genes are shown on the right, and the least stable genes on the left.

REFERENCES

1. Coussens PM, Verman N, Coussens MA, Elftman MD, McNulty AM. 2004. Cytokine gene expression in peripheral blood mononuclear cells and tissues of cattle infected with *Mycobacterium avium* subsp. paratuberculosis: evidence for an inherent proinflammatory gene expression pattern. Infect Immun 72:1409-22.
2. Cunha CW, Gailbreath KL, O'Toole D, Knowles DP, Schneider DA, White SN, Taus NS, Davies CJ, Davis WC, Li H. 2012. Ovine herpesvirus 2 infection in American bison: virus and host dynamics in the development of sheep-associated malignant catarrhal fever. Vet Microbiol 159:307-19.
3. Thacker TC, Palmer MV, Waters WR. 2007. Associations between cytokine gene expression and pathology in *Mycobacterium bovis* infected cattle. Vet Immunol Immunopathol 119:204-13.
4. Goossens K, Van Poucke M, Van Soom A, Vandesompele J, Van Zeveren A, Peelman LJ. 2005. Selection of reference genes for quantitative real-time PCR in bovine preimplantation embryos. BMC Dev Biol 5:27.
